# Computational structure-based design of antiviral peptides as potential protein–protein interaction inhibitors of rabies virus phosphoprotein and human LC8

**DOI:** 10.1016/j.heliyon.2024.e41520

**Published:** 2024-12-26

**Authors:** Saman Rahmati, Fatemeh Zandi, Khadijeh Ahmadi, Ahmad Adeli, Niloofar Rastegarpanah, Massoud Amanlou, Behrouz Vaziri

**Affiliations:** a Biotechnology Research Center, Pasteur Institute of Iran, Tehran, Iran; b Division of Oncological Sciences, Knight Cancer Institute, Oregon Health and Science University, Portland, OR, USA; c Department of Medical Biotechnology, School of Paramedicine, Bushehr University of Medical Sciences, Bushehr, Iran; d Department of Medicinal Chemistry, Faculty of Pharmacy, Tehran University of Medical Sciences, Tehran, Iran

**Keywords:** RABV phosphoprotein, LC8, Antiviral peptides, Docking, Molecular dynamics simulation

## Abstract

Rabies is a serious zoonotic disease caused by the rabies virus (RABV). Despite the successful development of vaccines and efforts made in drug discovery, rabies is incurable. Therefore, development of novel drugs is of interest to the scientific community. Antiviral peptides can be designed based on the known structures of viral proteins and their biological targets. Cytoplasmic dynein light chain LC8, one of the first identified host partners of RABV phosphoprotein (RABV P), is an essential factor for RABV transcription and replication. As part of the search for new potential drugs against rabies, we used structure-based drug design using the *in silico* tools. The binding site of LC8 with RABV P was used for peptide design. Four potential peptide inhibitors (Pep1-4) were selected, modeled, and docked with RABV P. The highest binding affinity was observed for the RABV P-Pep2 complex. Molecular dynamics (MD) simulations were performed and the stability of the peptides and complexes was confirmed. Finally, Pep2 can be used as a potential candidate for peptide-based antiviral therapy against RABV. The identified small peptides may prevent RABV infection based on the results of the current investigation. Further *in vitro* and *in vivo* studies are needed to confirm these results.

## 1 Introduction

Rabies is a fatal acute encephalomyelitis caused by the members of the genus Lyssavirus [1]. Rabies virus (RABV), the prototype virus of the genus, is the most successful lyssavirus species in terms of global distribution, abundance and progeny [1]. Despite the existence of effective vaccination, rabies is still a public health concern [2]. Unfortunately, there is no cure once clinical rabies develops mainly due to failure to receive valid post-exposure prophylaxis [3] so that about 59,000 human deaths are reported annually in the world [4]. Despite the remarkable progress made in drug discovery and development, no effective drugs have yet been approved for rabid patients [5] In this context, drug repurposing strategy using some drugs including ribavirin, interferon-alpha, ketamine, amantadine, favipiravir, and pyrimethamine has shown to be ineffective for rabies treatment [[6], [7], [8], [9], [10]]. However, few antiviral candidates such as λ-Carrageenan P32, clofazimine and its derivatives were suggested as promising inhibitors of RABV, worthy of further studies [5,11,12]. Besides, novel antiviral strategies employing oligonucleotide therapeutics, small molecules against host-virus protein-protein interactions (PPIs), and inhibitory peptides screened out from combinatorial peptide libraries, have been somewhat successful in RABV inhibition in related preliminary studies [[13], [14], [15], [16]]. Further investigation of the introduced inhibitory candidates may clarify their efficacy in rabies treatment. According to these data, the development of efficient therapeutic strategies against rabies which is emphasized by the WHO [9], should be pursued vigorously and intelligently, in order to save the lives of symptomatic patients.

Peptide-based antiviral therapeutics are newly emerging and seem promising [17]. Since the host-virus PPIs are essential for viral infection and pathogenesis [18], designing competitive peptides that interfere with these PPIs may serve as an efficient antiviral treatment [[19], [20], [21]]. Therefore, the selection of appropriate target (s) among host-virus PPIs for the design of inhibitory peptides, is an important factor in combating the viral infection. Since that most druggable PPIs are often dominated by hot segments (a continuous epitope that dominates the interaction), thus, designing peptides to target these PPIs could be a good inhibitory strategy [20].

RABV P, the viral RNA polymerase cofactor, is known for its central role in viral transcription and replication [22]. In addition, RABV P interacts with many host proteins in order to suppress host defense, regulate viral multiplication, and dysregulate mitochondrial function causing acute degenerative changes in neurons [23]. Thus, recent studies have targeted multifunctional RABV P in order to design antiviral agents [15,24,25]. Dynein light chain LC8 (10.3 kDa) is a subunit of the cytoplasmic dynein motor complex with the ability to bind number of different proteins through a common binding site [[26], [27], [28], [29]]. LC8 has been defined as a host interactor of RABV P and this binding is essential for rabies pathogenesis [30]. Residues 140–150 (SSEDKSTQTTG) of the central part of RABV P have been experimentally demonstrated to be the LC8-binding site [31]. It is evidenced that the RABV P-LC8 interaction has a functional role in the regulation of viral primary transcription. Inhibition of this binding did not inhibit RABV entry into the central nervous system (CNS) of a mouse model but significantly suppressed the viral transcription and replication in the CNS, thereby inhibiting the onset of clinical rabies [30]. Considering the importance of RABV P-LC8 complex, this PPI was identified as a candidate therapeutic target in the present study. We have, herein, designed structure-based peptides based on a common LC8 binding site with full-length RABV P (isoform P), which would hypothetically compete with LC8 for RABV P binding. The common LC8 binding site for its ligands (obtained by LC8-ligand complex crystal structure analysis) together with the observed LC8 binding region of the docked RABV P-LC8 complex after MD simulation, were used to design antiviral peptides. The binding affinity and stability of the RABV P-peptide complexes were then evaluated by molecular docking and MD studies. As a result, one of the designed antiviral peptides revealed the highest binding affinity to RABV P, almost similar to the original LC8 protein compared. Therefore, this peptide may have promising potential for further development.

## 2 Materials and methods

### 2.1 RABV strain CVS-11 P protein homology modeling and evaluation of the modeled 3D structure

The P isoform of the RABV phosphoprotein, the full-length form of phosphoprotein (297 a.a), acts as a cofactor of the viral RNA polymerase, while N-terminally truncated isoforms (P2-P5) do not have cofactor activity [32]. Considering the importance of the LC8 binding domain of the RABV P protein for efficient viral RNA polymerase activity that is consistent with its role as the viral RNA polymerase cofactor [30], the full-length RABV P was used to design potential antiviral peptides. The protein sequence of the full-length RABV P belonging to the pathogenic CVS-11 strain of RABV (Accession No. P22363) was retrieved from the UniProt Knowledgebase (UniProtKB). The three dimensional (3D) structure of this protein was modeled using the I-TASSER web server (http://zhang.bioinformatics.ku.edu/I-TASSER), MODELLER (https://salilab.org/modeller/), and AlphaFold2 (https://github.com/sokrypton/ColabFold) servers. I-TASSER predicts the protein structure and function in general steps including threading template identification, iterative structure assembly simulation, model selection and refinement, and structure-based function annotation [33] The C-score which is a confidence score for estimating the quality of predicted models, is calculated according to the significance of the threading template alignments and the convergence parameters of the structure assembly simulations [34]. MODELLER uses comparative or homology modeling to predict protein structures by aligning the target sequence with known template structures. It builds a 3D model by generating and optimizing spatial restraints from this alignment. MODELLER refines the model using various optimization techniques and evaluates it with physics-based energy functions, considering factors like bond lengths, angles, and non-bonded interactions [35]. AlphaFold, developed by Google DeepMind, uses AI and deep learning to predict protein structures from amino acid sequences. Its neural network, called Evoformers, processes these sequences to predict the 3D coordinates of all heavy atoms in the protein. AlphaFold iteratively refines the structure and provides confidence metrics to indicate the reliability of different parts of the predicted structure [36]. PDB structures related to RABV P (CVS-11 strain) including 1vyi, 8b8v, and 8fuq were used as the templates by MODELLER and AlphaFold2 servers in order to model the RABV P. After modeling, all three models built using the I-TASSER, MODELLER, and AlphaFold2 servers were compared in terms of structure quality. Evaluation of the 3D models quality was performed using ERRAT (https://www.doe-mbi.ucla.edu/errat/webservers) [37], ProSA (https://prosa.services.came.sbg.ac) [38], and MolProbity (http://molprobity.biochem.duke.edu) [39] web servers. In ERRAT analysis, incorrect regions of protein structures are recognized according to errors leading to random distributions of atoms that can be distinguished from correct distributions [37,40]. The ERRAT overall quality factor value for high resolution structures is around 95 % or higher. The results of ProSA web tool are indicated as Z-score and residue energy plot. The Z-score, which indicates overall model quality is displayed in a plot containing the Z-scores of all experimentally determined protein chains in PDB. The Z-score of the query protein should be within the range of scores of native proteins of similar size. Incorrect structures are predicted by Z scores outside this range. In residue energy plots, positive values simply indicate problematic regions of the model [41]. MolProbity tool checks the stereo-chemical problem of the tertiary protein structure. The clashscore, rotamer, and Ramachandran evaluations are combined to make the MolProbity score which is indicator of model quality [42,43]. Experimental structures are expected to have MolProbity scores below 2 and scores around 1 are desirable for high-quality structures [44]. Finally, the modeled RABV P structure with the highest quality was selected for subsequent studies. The binding site residues of RABV P include Glu142, Asp143, Lys144, Ser145, Thr146, Glu147, and Thr148 [31,45]. Residues Asp143 and Gln147 of this sequence have been identified as hotspots necessary for binding to LC8 [31,45].

### 2.2 3D structure and ligand-binding site retrieval of LC8

LC8 binds to all its ligands containing KXTQT or GIQVD sequence fingerprints (LC8 binding motifs) through a common binding site [28,29]. Thus, the 3D crystal structure of human LC8 in complex with one of its ligands, the Nek9 peptide (PDB ID: 3ZKF), which contains KXTQT sequence like RABV P, was obtained from the Protein Data Bank (PDB) in order to define the 3D structure and ligand-binding region of human LC8 [46]. Interactions between LC8 and Nek9 were observed using PyMOL (version 1.8) and LIGPLOT (version 2.2.8) [47]. The binding site residues of human LC8 include Asn10, Arg60, Asn61, Phe62, Gly63, Ser64, Tyr65, Val66, Thr67, His68, Glu69, Thr70, Phe73, Tyr75, Try77, Gly79, Ala82, Ser88, which were observed using the LIGPLOT program based on the 3ZKF protein complex [46].

### 2.3 Molecular dynamics simulation studies of modeled RABV P & LC8

MD simulation of the modeled RABV P was performed using GROMACS 2020.3 with the AMBER force field (amber99sb-ildn) in order to determine the stability and conformational behavior of this protein in a dynamic environment. In addition, although the crystal structure of human LC8 was available, an MD simulation of this molecule was performed to balance the conditions of the two protein molecules for the docking process. The RABV P and LC8 structures were placed in a simulation chamber filled with TIP3P water molecules. The system was neutralized with sodium and chlorine ions. Periodic boundary conditions were applied in all directions. Electrostatic interaction simulations were performed through the particle mesh Ewald method [48,49]. The electrostatic and Van der Waals cut-off of 12 Å and 14 Å were used for the process. The SHAKE method [50] was used for the covalent bonds involving hydrogen.

The protein system was then energy minimized using the steepest descent algorithm with a tolerance of 1000 kJ/mol/nm. After convergence, the system was run through NVT ensemble (constant number of particles, volume and temperature) MD simulation for 20 ps. The MD simulation was performed using the NPT ensemble (constant number of particles, pressure and temperature) in a periodic boundary condition. The pressure and temperature of the system were kept constant at 1 bar and 300 K with a coupling time of τp = 0.5 ps, and τT = 0.1 ps, respectively using Berendsen barostat and thermostat. Finally, the MD simulations were extended at constant pressure and temperature for 100 ns for each protein structure under study. The simulated system was then analyzed for trajectories such as root mean square deviation (RMSD), root mean square fluctuation (RMSF), radius of gyration (Rg), solvent accessible surface area (SASA), and the secondary structure (DSSP) using inbuilt programs in the GROMACS package [51,52].

### 2.4 Molecular docking of RABV P and LC8, followed by MD simulation studies of the complex

The protein-protein molecular binding between the RABV P and the LC8 has been studied using the HADDOCK2.2 and PRODIGY web servers [53]. The 3D structures of two proteins after MD simulation were used as the inputs of HADDOCK tool and binding and non-binding site residues of each protein were dictated to web server. The best models of the predicted complexes were chosen based on the interacting residues, type of interactions (mainly hydrogen bonds, hydrophobic and electrostatic interactions), and HADDOCK score. In this regard, LIGPLOT was used to analyze and represent the interaction site residues together with hydrogen, hydrophobic and electrostatic bonds within each docked model. Finally, the predicted model with the lowest HADDOCK score which represented the highest number of interaction site residues and highest interaction bonds was introduced as the best docked model and its binding affinity and equilibrium dissociation constant (kd) were predicted by the PRODIGY web server (https://bianca.science.uu.nl/prodigy/) [54]. The predicted complex's stability, structural changes, and binding free energy were then assessed by MD simulation trajectory for 100 ns as described before. The docked complex was evaluated by RMSD, RMSF, Rg, SASA, and Molecular Mechanics Poisson-Boltzmann Surface Area (MM/PBSA) analysis using GROMACS software. Analysis of the Molecular docking and MD simulation results were done using VMD 1.9.3 software. The obtained results were visualized with the aid of PyMOL and Discovery Studio 4.1 Client. Microsoft Office Excel 2016 was applied to represent all graphs.

### 2.5 Peptide designing and evaluation

Visual inspection of the LC8 binding region of the docked RABV P-LC8 complex after MD simulation study together with derived information about the LC8 binding site of the Human LC8- Nek9 peptide (PDB ID: 3ZKF) [46] provided the basis for the design of structure-based antiviral peptides. To design peptides, the protein-protein interface of MD simulated RABV P-LC8 complex was analyzed using LIGPLOT and PPCheck server. PPCheck (http://caps.ncbs.res.in/ppcheck/) [55] ascribes pseudoenergies to protein-protein interface and Graph theoretical parameters are employed for all the interface residues to predict hotspots.

The 3D structure or folding of the designed antiviral peptides was predicted by the PEP-FOLD3 web server (http://bioserv.rpbs.univ-paris-diderot.fr/services/PEP-FOLD3) which uses a Hidden Markov Model suboptimal sampling algorithm [56]. The sequence information of each peptide was used to estimate the peptide folding. The tertiary structure quality of the modeled peptides was assessed using the Ramachandran plot calculated by MolProbity [39]. The antigenicity of each peptide was characterized by the Proinflam web server **(**http://metagenomics.iiserb.ac.in/proinflam/) [57] which is designed to predict the proinflammatory antigenicity of peptides. The toxicity of peptides was characterized using the ToxinPred we bserver (https://bio.tools/toxinpred) [58]. The Peptide Solubility Calculator tool (https://pepcalc.com/) was used to assess the solubility of the peptides [59]. MD simulations of the modeled peptides were performed to assess the stability of the structures. The structures were placed in a simulation chamber containing TIP3P water. The system was then neutralized by the addition of a specific concentration of Na+ and Cl-ions. Periodic boundary conditions were applied in all directions. The Ewald particle mesh method was used to simulate electrostatic interactions. After convergence, the system was run through an NVT ensemble (constant number of particles, constant volume, and constant temperature) MD simulation for 20 ps. The MD simulation used the NPT ensemble (constant number of particles, pressure, and temperature) in a periodic boundary condition. MD simulation of each peptide structure was run for 100 ns at continuous pressure and temperature. Trajectories such as RMSD, RMSF and Rg were then analyzed in the GROMACS package for the simulated system.

### 2.6 Molecular docking of RABV P and peptides, and MD simulation studies of the complexes

The 3D structures of the RABV P and four peptides following MD simulation were used to perform information-driven docking by the HADDOCK tool. The best model of each predicted docked RABV P-peptide (Pep1-4) complex was selected, and the binding affinity (equilibrium dissociation constants: Kd) for each complex was predicted by the PRODIGY web server as described above. The docked RABV P-peptide molecules and their conformational stability were then evaluated by MD simulations for 100 ns in GROMACS software and simulation trajectories such as RMSD, Rg, RMSF, and SASA were studied.

### 2.7 Calculations of binding free energy

The binding free energies of the protein-peptide complexes were calculated by the Molecular Mechanics-Poisson-Boltzmann Solvent-Accessible Surface Area, MM/PBSA method [60]. The total binding free energies were measured using MM/PBSA script and the contribution of electrostatic energy, van der Waals energy, apolar and polar solvation energy.

## 3 Results

### 3.1 Homology modeled RABV P and structural validation

**Fig. 1:**
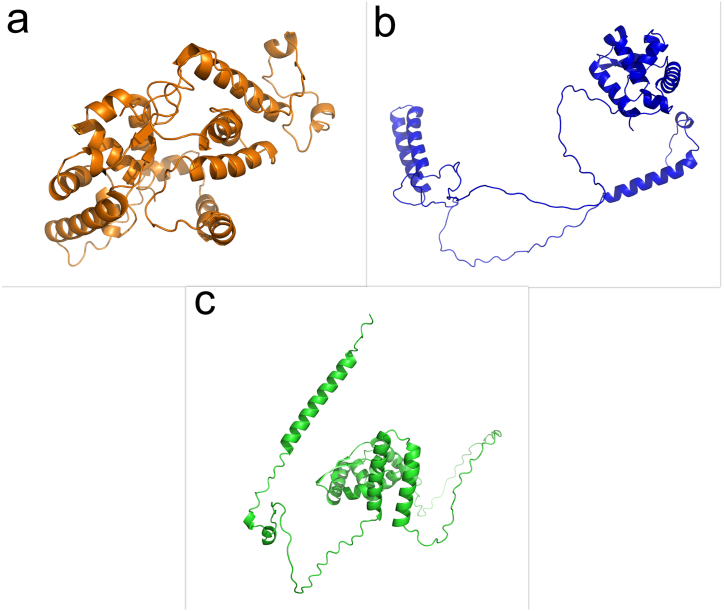
Schematic representation of the modeled protein by (a) I-TASSER, (b) MODELLER, and (c) AlphaFold2.

The 3D structure of the RABV P protein was predicted by homology modeling using I-TASSER, MODELLER and, AlphaFold2 servers (Fig. 1a–c). In I-TASSER homology modeling, the target sequence is threaded to search for the possible folds using a representative PDB structure library [53]. From the five predicted models, the model with the best C-score of −3.54 was selected with an estimated accuracy of 0.33 ± 0.11 (TM-Score) and 14.8 ± 3.6A (RMSD). Since the C-score is typically in the range of (-5- 2), the RABV P predicted 3D model was considered as a confident model for further analysis. The DOPE model score is designed to select the best structure from a set of MODELLER models. The normalized DOPE score is used to accurately select a model from the 5 MODELLER models. The most negative DOPE score is the best model. Negative normalized DOPE scores of −1 or less will likely correspond to models with the correct fold [61]. In this study, all models created using the MODELLER server had a DOPE score greater than −1. AlphaFold2 uses two key metrics, pLDDT (per-residue confidence score) and pTM (predicted TM score), to assess the confidence of its predicted protein structure. High pLDDT scores (>80) indicate a high degree of accuracy in the residue structure, and low pLDDT scores (<50) may indicate that the residues are located in inherently disordered regions of the protein [62]. The PTM score ranges from 0 to 1. A score closer to 1 has higher confidence in the accuracy of the global structure, indicating a likely correct fold, and a score closer to 0 has lower confidence in the global structure, indicating a potentially incorrect topology [63]. The best model built with Alphafold 2 had pLDDT = 58.9 and pTM = 0.349. Validation of the predicted structure quality by all three servers was then performed using web servers including ERRAT, ProSA, and MolProbity. The ERRAT scores for the structure modeled by I-TASSER, AlphaFold2, and MODELLER were 90.311, 91.589 and 74.194, respectively. ProSA-web used an energy plot and a Z-score to examine the three-dimensional models. For all three models the Z-score was at the desired point. The Z-score of the protein selected by I-TASSER, AlphaFold2, and MODELLER was −5.47, −3.78 and −5.16, respectively (Supplementary Fig. S1). According to the MolProbity analysis, the protein predicted by I-TASSER had a clash score of 6.62 (89 % best of all structures), the protein predicted by AlphaFold2 had a clash score of 11.97 (63 % best of all structures), and the structure modeled by MODELLER had a clash score of 37.01 (9 % best of all structures). Finally, the evaluation of the third structure showed that the structural features predicted by I-TASSER and AlphaFold2 were of higher quality than the structure predicted by MODELLER. Both the I-TASSER and AlphaFold2 models had acceptable ERRAT and Z-score values, but as the clash score of the I-TASSER model (6.62) was more acceptable than that of the Alphafold2 model (11.97), and its structure was of higher quality, it was selected for further study. The I-TASSER server used PDB structures; 1vyiA, 8ok8A, 7t5gA, 8ok4A, 1vyi, 7ogtB and 8b3bA as the templates to generate the RABV P protein structure.

### 3.2 MD simulation of the modeled RABV P

**Fig. 2:**
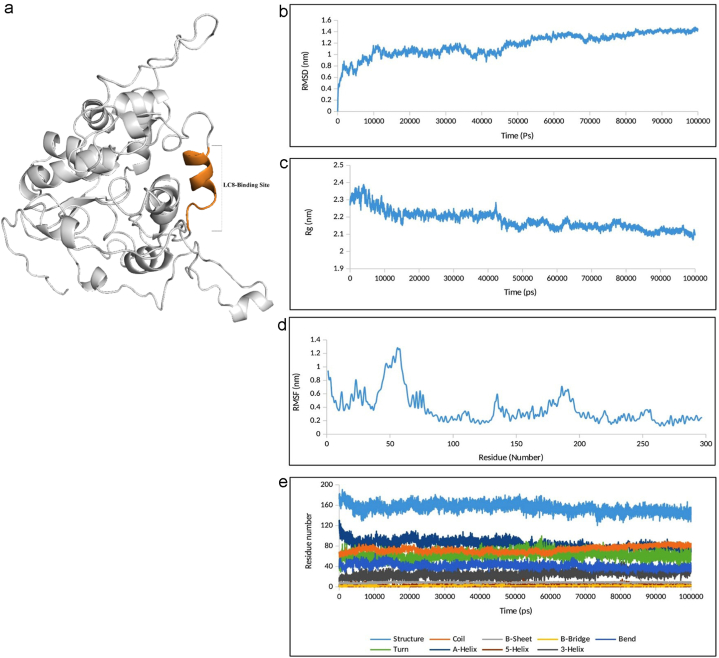
The tertiary model and stability assessment of the MD simulated RABV P. (a) The post MD 3D structure of RABV P with shown LC8-binding site. (b) In MD simulation analysis of the modeled RABV P in 100ns, The RMSD plot shows the achievement of the stability from 54 ns. (c) The Rg plot analysis represents that the RABV P reached the stable and compact conformation around 45 ns. (d) The higher RMSF value especially within 46-59th residues corresponding with the loop regions could be seen in the RMSF plot. (e) The plot of the secondary structure changes of RABV P exhibits low conformational changes of RABV P structure during the simulation.

To evaluate the stability and dynamics of the modeled RABV P, MD simulation was performed for 100 ns. The RMSD plot analysis of the modeled RABV P showed that the structure reached a steady state in about 54 ns of the simulation with an RMSD value between 1.2 and 1.4 nm. The Rg plot analysis represented that the structure reached a steady state in about 45 ns of the simulation with an Rg value between 2.1 and 2.2 nm, indicating that the RABV P reached the stable and compact conformation during simulation. The structural changes in RABV P after MD simulation were characterized by calculating RMSF value. The RMSF plot showed that the primary parts of the N-terminal region of RABV P especially within 46–59 residues, have a higher RMSF value than the C-terminal region which could be due to the higher number of loop regions in this region compared to the rest of the structure. The stability of the secondary structure during the MD simulation was analyzed and the result exhibited low conformational changes of the RABV P structure during the simulation. The quality of the modeled RABV P after MD simulation was also investigated. Fortunately, the calculated scores for the ProSA, ERRAT, and MolProbity tools were considerably improved to −7.81, 91.797 %, and 1.97, respectively and residue energies for the whole structure were entirely negative indicating the high quality of the model **(**Supplementary Fig. S2**).** The 3D model and stability assessment of the MD simulated RABV P, is shown in Fig. 2a–e**.** Overall, the RABV P structure obtained by MD simulation showed acceptable quality and stability, which was used for the next molecular docking analysis.

### 3.3 Molecular docking and MD simulation studies of the RABV P-LC8 complex

**Fig. 3:**
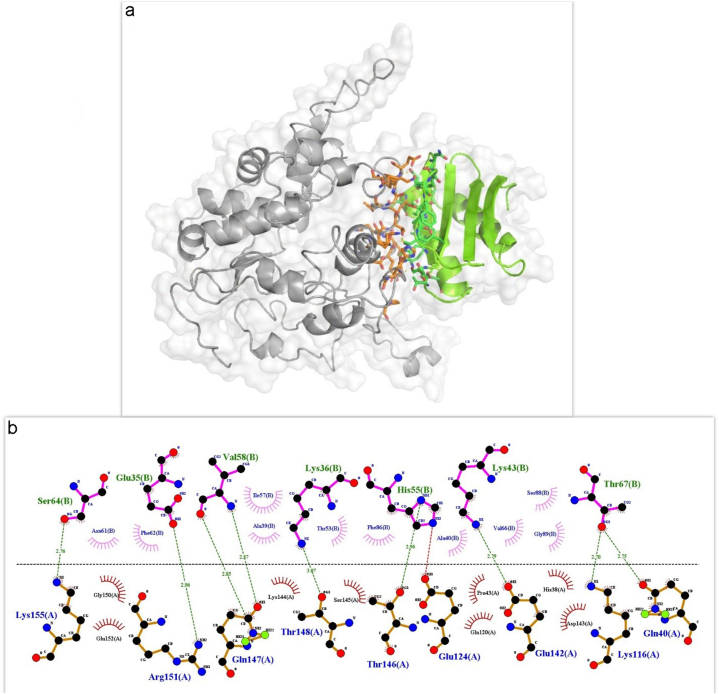
Molecular docking study of the P-LC8 complex. (a) The best docked model of RABV P-LC8 complex representing polar contacts in protein interface derived from PyMOL. The 3D structure of RABV P (grey) bound with LC8 (green). Residues of RABV P and LC8 involved in the interaction were colored in orange and dark green, respectively. (b) Representation of the interactions between RABV P protein and LC8 by LIGPLOT diagram. The amino acid residues at RABV P interface and LC8 interface involved in hydrophobic interactions are shown as brown and pink spoked arcs, respectively. Side chains of amino acid residues involved in hydrogen bond interactions are shown as ball-and-stick models. Hydrogen bond is shown by green dotted line.

The HADDOCK and PRODIGY servers were used to study the protein-protein molecular binding between RABV P and LC8. We considered the lowest energy, the lowest Kd, and the highest number of amino acids involved in hydrogen bonding and hydrophobicity as essential criteria for selecting the strongest complex. The results showed a strong interaction between the RABV P and the LC8 (Table 1). The interactions of the RABV P-LC8 complex were observed using the PyMOL and LIGPLOT programs (Fig. 3a and b).

**Table 1:** Molecular docking and PRODIGY analyses of RABV P-LC8 complex.

Docked proteins	HADDOCK score	Binding affinity (ΔG) in kcal/mol (by PRODIGY)	Dissociation constant (Kd) in Molar (by PRODIGY)	Interacting amino acids
RABV P-LC8	−74.4 ±2.8	−9.1	2.1E-07	*RABV P:*His38, Gln40, Pro43, Lys116, Gln120, Glu142, Asp143, Lys144, Ser145, Thr146, Gln147, Thr148, Gly150, Arg151, Glu152, lys155
				*LC8:* Glu35, Lys36, Ala39, Ala40, Thr53, His55, Ile57, Val58, Asn61, Phe62, Ser64, Val66, Thr67, Phe86, Ser88, Gly89, Lys43

**Fig. 4:**
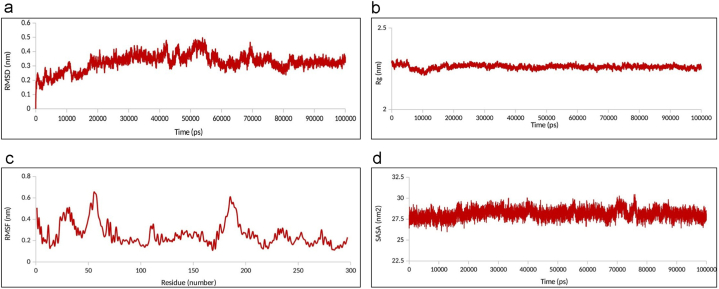
The MD simulation result of the RABV P-LC8 complex in 100 ns. (a) The RMSD plot indicates that the complex became stable around 80ns of simulation with RMSD value between 0.3 and 0.4 nm. (b) In the Rg plot, compactness and relaxation of the complex could be seen at 10 ns of the simulation. (c) The RMSF plot of the complex shows great improvement in stability compared to the free RABV P, especially in the primary parts of the N-terminal region of RABV P. (d) Based on SASA plot analysis, the overall exposure to the solvent area remained fairly unchanged for the complex with the SASA value around 28 nm^2^ up to 100 ns.

In the next step, MD simulation of the RABV P-LC8 complex was performed for 100 ns. The trajectory analysis is depicted in Fig. 4a–d. The RMSD plot analysis showed that the complex reached stability with fluctuations of less than 0.3 nm. The Rg plot indicated that the complex became compact and relaxed after 10 ns of the simulation. The RMSF plot revealed that the stability of RABV P in the complex was greatly improved compared to the free RABV P, especially in the primary parts of the N-terminal region of RABV P which showed fluctuations in the free form of RABV P. SASA plot analysis showed that the SASA value for the complex was around 28 nm^2^ up to 100 ns for the complex, which indicates that the overall exposure to the solvent area remained fairly unchanged.

**Fig. 5:**
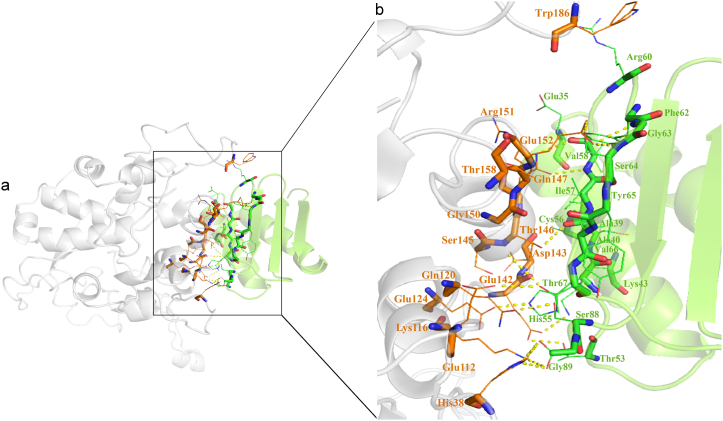
Structural analysis of the RABV P-LC8 complex after MD simulation. (a) The 3D structure of RABV P (grey) bound with LC8 (green), Residues of RABV P and LC8 involved in the interaction were colored in orange and dark green, respectively. (b) Close up view of interacting interface between RABV P and LC8. The crucial residues are presented as sticks (orange/RABV P protein, dark green/LC8). Hydrogen bonds between chains are shown based on LIGPLOT diagram.

Analysis of the protein complex interface extracted from the steady state of the simulation showed that the binding pocket of RABV P-LC8 was largely preserved compared to it before simulation (Fig. 5a, b and Table 2). The LIGPLOT program revealed that most of the key residues of the LC8-binding site of RABV P [31,45] and the ligand binding site of human LC8 [46] were present in the complex interface (protein-protein interface) after simulation. Glu142, Asp143 (hot spot), Ser145, Thr146, Gln147 (hot spot), and Gly150 are the key interacting amino acids of RABV P and Arg60, Phe62, Gly63, Ser64, Tyr65, Val66, Thr67, and Ser88 are the key interacting amino acids of LC8 which are also present in the interface of the MD simulated complex.

**Table 2:** Residues in the interaction of RABV P-LC8 complex after MD simulation.

	**RABV P**	**LC8**
Residues in the interaction of RABV P-LC8^a^	His38, Glu112, Lys116, Gln120, Glu124, Glu142, Asp143, Ser145, Thr146, Gln147, Gly150, Arg151, Glu152, Thr158, Trp186	Glu35, Ala39, Ala40, Lys43, Thr53, His55, Cys56, Ile57, Val58, Arg60, Phe62, Gly63, Ser64, Tyr65, Val66, Thr67, Ser88, Gly89,

a The underlined amino acids are common amino acids in the RABV P-LC8 complex before and after MD simulation.

### 3.4 Peptide designing and evaluation

**Fig. 6:**
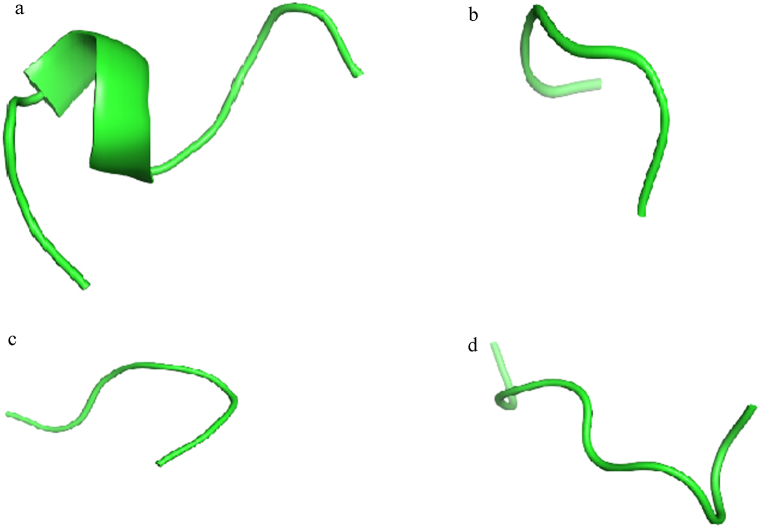
The best four peptide based on binding affinity and binding energy in docking program; (a) Pep1, (b) Pep2, (c) Pep3, (d) Pep4 peptide molecules.

**Fig. 7:**
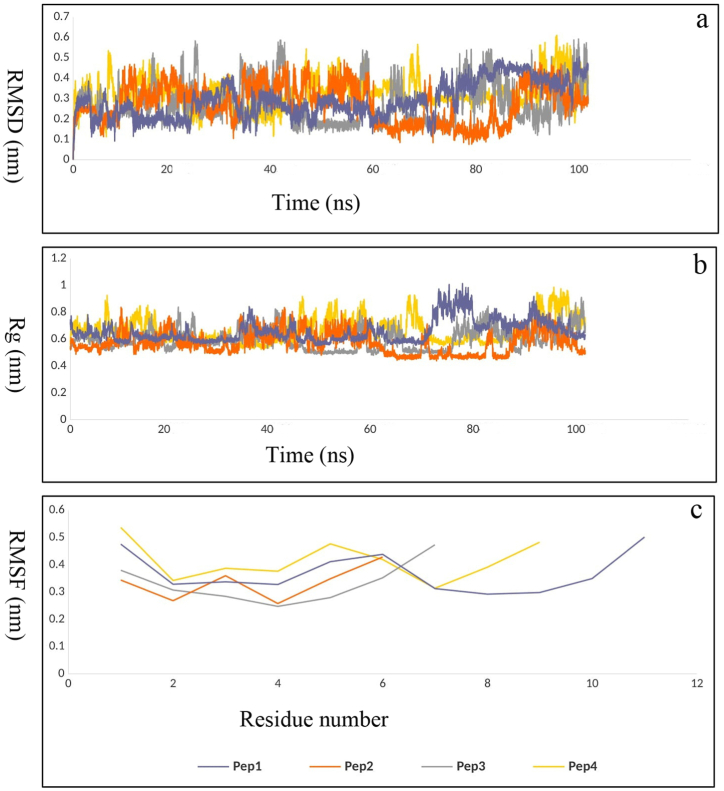
The MD simulation result of the modeled peptides in 100 ns. (a) RMSD results of the modeled peptides in unit time (ns). (b) Rg results of the modeled peptides per unit time. (c) RMSF results of the modeled peptides.

The analysis of amino acids involved in the interaction between RABV P and LC8 after MD simulation showed that residues including Arg60, Phe62, Gly63, Ser64, Tyr65, Val66, and Thr67, which belong to the eleven consecutive amino acid containing segment (residues: 60–70) of the LC8 binding region [28,29,46], are present in this interface. This β-sheet structured segment is a continuous epitope that contributes many of the key residues (residues: 60–70) belonging to the LC8 binding region. Therefore, it seems to be a hot segment in this interaction. On the other hand, a report of studying the docked complex of the modeled RABV P and LC8 showed that P peptide (residues: 140–150) from RABV P binds mainly to this β-sheet structured segment and Asp143, hot spot residue of P peptide, makes hydrogen bonds with this segment [31]. All these features make this eleven consecutive amino acid containing segment (residues: 60–70) of LC8 binding region suitable for precursors of peptide PPI inhibitors. The entire segment (residues: 60–70) was considered as the base peptide (Pep1) 60-RNFGSYVTHET-70 and three sequence fragments from this segment were selected as peptides: (Pep2) 60-RNFGSY-65, (Pep3) 60-RNFGSY--H-68, and (Pep4) 60-RNFGSY--HET-70. The peptide model structure for each designed peptide derived from the PEP-FOLD3 is presented in Fig. 6a–d. As shown in Table 3, the modeled peptides are of high quality. It was predicted that the Pep1, Pep2, and Pep4 peptides would have good solubility in water and Pep3 would have poor solubility in water. The Ramachandran plot showed that almost all model residues were in the favored region and no residues were detected in the outlier region, indicating that the backbone ϕ/ψ torsion angles were accurately constructed for the all models (Supplementary Fig. S3). The proinflammatory antigenicity and toxicity of the peptides were evaluated using the Proinflam and ToxinPred tools, respectively. Their proinflammatory antigenicity situation was negative and they were characterized as non-toxic **(**Supplementary Table S1**).** In order to check the stability of the modeled peptide structures, MD simulations up to 100 ns were also carried out. According to Fig. 7a–c, the RMSD, Rg and RMSF fluctuations of the peptides show that all four structures have the required stability with fluctuations of less than 0.3 nm during the simulation. In the next step, the peptide structures were used to dock against the RABV P and MD simulation studies of the complexes were performed.

**Table 3:** Quality details of peptide models generated by PEP-FOLD3.

**No.**	**sOPEP**	**avg**	**gdt**	**max**	**q**	**tm**
Pep1	−5.78351	0.603	0.761	0.656	0.546	0.451
Pep2	−2.55802	0.911	0.979	0.956	0.913	0.795
Pep3	−2.99607	0.817	0.939	0.882	0.787	0.661
Pep4	−4.52067	0.728	0.878	0.809	0.703	0.520

### 3.5 Molecular docking and MD simulation studies of RABV P-peptide complexes

**Fig. 8:**
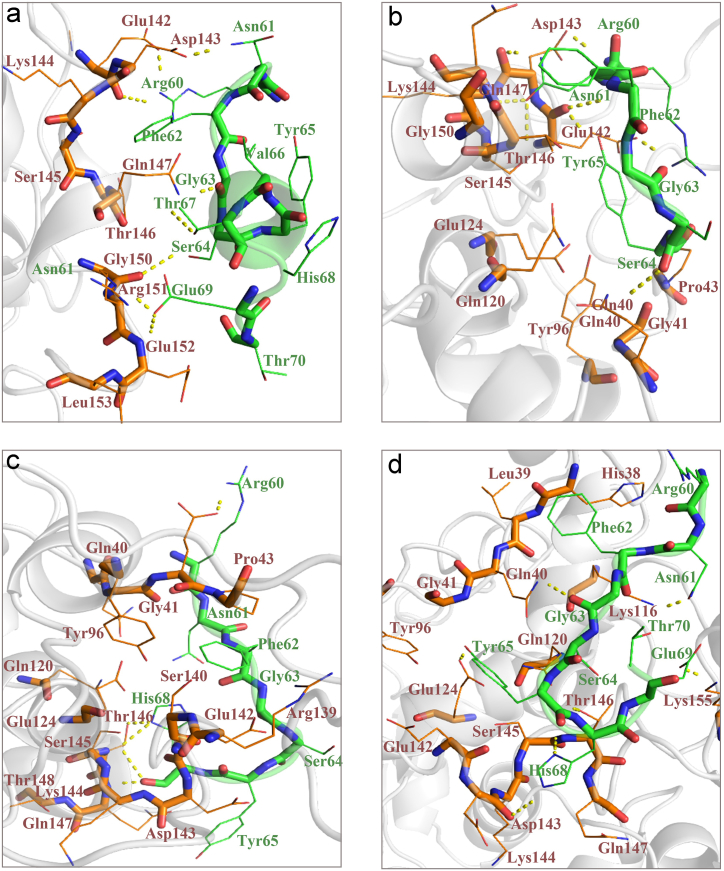
Protein interfaces of the (a) RABV P-Pep1, (b) RABV P-Pep2, (c) RABV P-Pep3, and (d) RABV P-Pep4 complexes after molecular docking including polar contacts derived from PyMOL.

To determine the best molecular interaction orientation between the MD simulated peptides (Pep1-4) and RABV P, information-driven docking was performed using the HADDOCK tool. The models with the lowest energy scores with the highest number of interaction site residues (experimentally determined for RABV P) and highest interaction bonds (represented by LIGPLOT), were selected as the best docked complexes. The HADDOCK score, binding affinity, and binding energy of the best docked model of RABV P-peptide (Pep1-4) complexes are presented in Table 4. The binding affinity calculation by the PRODIGY web server showed that the RABV P-Pep1 complex had higher binding affinity and lower binding energy compared with the other RABV P-peptide complexes. The binding interfaces of RABV P-peptide (Pep1-4) complexes derived from PyMOL are shown in Fig. 8a–d.

**Table 4:** Molecular docking and PRODIGY analyses of RABV P- peptide (Pep1-4) complexes.

**Docked complex**	**HADDOCK score**	**Binding affinity (ΔG) in kcal/mol** (by PRODIGY)	Dissociation constant (K_d_) in Molar (by PRODIGY)	Interacting amino acids
RABV P-Pep1	−68.7 ±6.5	−6.5	1.6E-05	*RABV P*: Glu142, Asp143, Lys144, Ser145, Thr146, Gln147, Gly150, Arg151, Glu152
				*Pep1:* Arg60, Asn61, Phe62, Gly63, Ser64, Val66, Thr67, Glu69, Thr70
RABV P-Pep2	−52.9 ±7.1	−5.7	6.5E-05	*RABV P:* Gln40, Gly41, Pro43, Gln120, Glu124, Glu142, Asp143, Lys144, Ser145, Thr146, Gln147, Gly150
				*Pep2:* Arg60, Asn61, Phe62, Gly63, Ser64, Tyr65
RABV P-Pep3	−54.6 ±6.8	−6.0	4.2E-05	*RABV P:* Gln40, Glu42, Pro43, Tyr96, Gln120, Glu124, Glu142, Asp143, Lys144, Ser145, Thr146, Gln147
				*PeP3:* Arg60, Asn61, Phe62, Gly63, Ser64, Tyr65, His68
RABV P-Pep4	−75.4 ±6.5	−6.2	2.9E-05	*RABV P:* His38, Gln40, Lys116, Gln120, Glu124, Glu142, Asp143, Lys144, Ser145, Thr146, Gln147, lys155
				*PeP4:* Arg60, Asn61, Phe62, Gly63, Ser64, Tyr65, His68, Glu69, Thr70

**Fig. 9:**
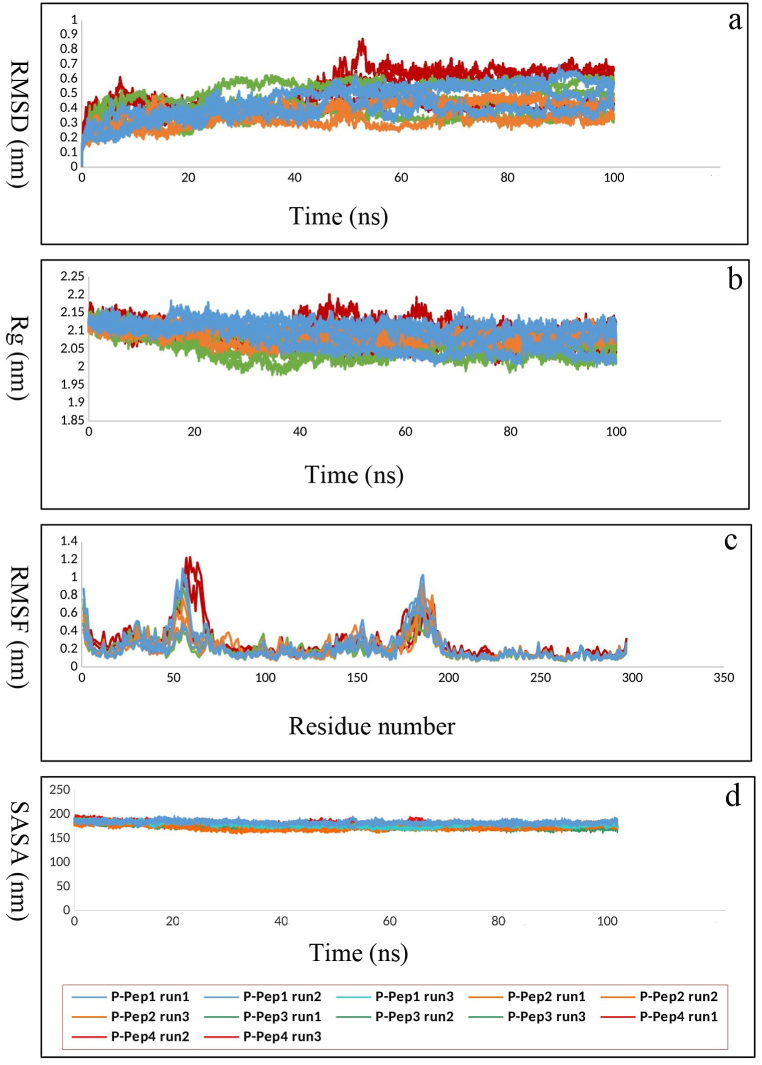
MD simulation of the designed peptides (Pep1-4) against RABV P with three replicates. (a) In RMSD plot, of RABV P-RABV P-Pep2, and RABV P-Pep3 showed a trend of uniform steady state during 100 ns of simulation whereas the RABV P-Pep1 and RABV P-Pep4 complex achieved stability after 50 ns for the rest of the simulation time. (b) Based on Rg plot, RABV P-Pep1, RABV P-Pep2, RABV-Pep3, and RABV P-Pep4 showed acceptable compactness during whole simulation. (c) The RMSF of RABV P-peptide complexes indicates that the primary parts of the RABV P N-terminal (especially within 50–70 residues) and C-terminal (especially within 180–190 residues) in complex with Pep1 and Pep4 have less stability and more flexibility compared to other complexes which mostly belong to the loop regions. (d) In SASA plot analysis, RABV P-Pep1, RABV P-Pep2, RABV P-Pep3, and RABV P-Pep4 complexes remained stable during simulation.

For further analysis, molecular dynamics simulations of docked RABV P-peptide (Pep1-4) complexes were performed with three replicates to investigate the structural stability of each complex. As shown in Fig. 9a the backbone RMSD plot of RABVP-Pep2, and RABV P-Pep3 indicated a trend of uniform steady state during 100 ns of simulation, whereas the RABV P-Pep1 and RABV P-Pep4 complexes exhibited minor RMSD fluctuations up to 50 ns, after which the complexes achieved stability throghout the rest of the simulation time. For the RABV P-Pep1 complex, the average RMSD of the first, second and third runs were 0.38, 0.44 and 0.46 nm, respectively. The reproducibility of the results for the RABV P-Pep2 complex was confirmed with RMSD averages of 0.31, 0.38, and 0.38 nm. For the RABV P-Pep3 complex, RMSD averages were 0.53, 0.41, and 0.32 nm; despite slight differences, all three runs demonstrated complex stability. The RABV P-Pep4 complex results also showed reproducibility, with RMSD averages of 0.39, 0.51, and 0.54 nm, indicating stability across all three MD simulation runs. These results indicate overall structural stability for all the four RABV P-peptide complexes. To evaluate the folding and compactness of the RABV P-peptide complexes the Rg plot was examined. The results of Rg for all four RABV P-Pep complexes showed the stability of the complexes during the simulations. Also, the triple reproducibility of RABV P-Pep1 (2.1, 2.1 and 2.06 nm), RABV P-Pep2 (2.09, 2.07 and 2.09 nm), RABV P-Pep3 (2.04, 2.08 and 2.05 nm) and RABV P-Pep4 (2.06, 2.09 and 2.1 nm) confirmed the stability of the complexes during the simulations. The RABV P-Pep4 complex fluctuated less than 0.1 nm in 40–60 ns, and overall the complex was stable (Fig. 9b).

To understand the flexibility across the amino acid region of the RABV P protein in complex with the four peptides, the RMSF of the RABV P-peptide complexes were calculated.

Most of the fluctuations correspond to the primary parts of the RABV P N-terminal (especially within 50–70 residues) and C-terminal (especially within 180–190 residues) which mostly belong to the loop regions. As shown in Fig. 9c, RABV P complexed with Pep1 and Pep4 exhibited greater changes in these two fluctuation regions compared to RABV P in complex with Pep2 and Pep3. This observation suggests that these two regions of the RABV P protein sequence are less stable and more flexible when complexed with Pep1 and Pep4 than with other peptides.

Changes in the solvent accessible surface area of the RABV P-peptide complexes were evaluated through SASA plot analysis. The RABV P-Pep1, RABV P-Pep2, RABV P-Pep3, and RABV P-Pep4 complexes showed similar SASA trends throughout the simulation time with no fluctuation, indicating no change in the exposure to the solvent area for these complexes (Fig. 9d). The 25, 50, 75, and 100 ns snapshots, which check the state of the complex during the simulation, show that the structure of the RABV P-peptide complexes and the interaction site of the RABV P-protein are stable during the simulation (Supplementary Figure S4).

**Fig. 10:**
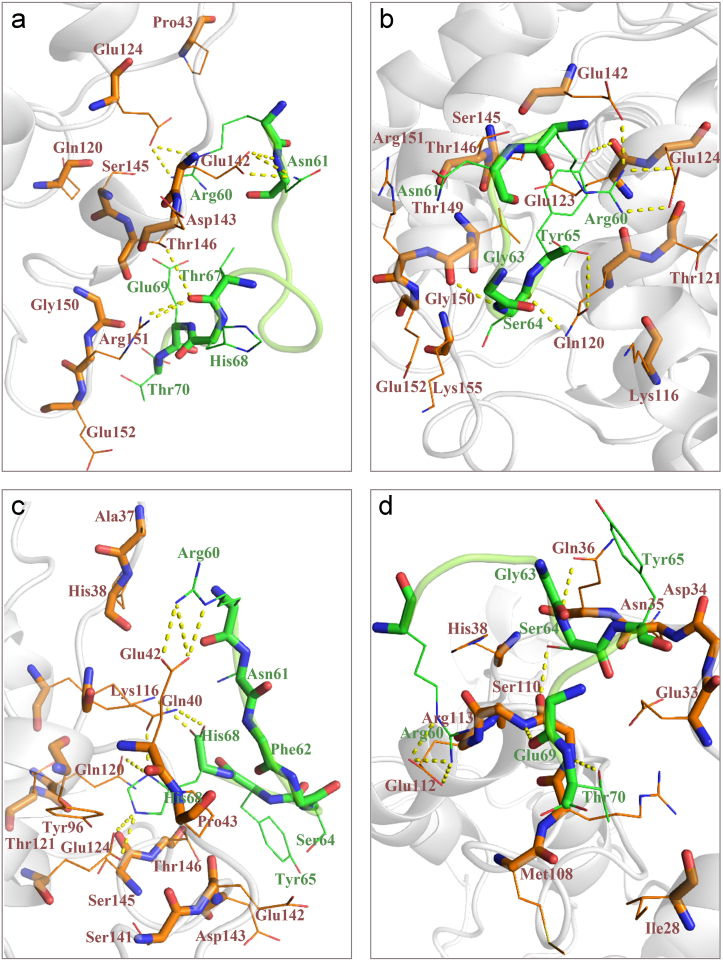
Protein interfaces of the RABV P-peptide. (a) RABV P-Pep1, (b) RABV P-Pep2, (c) RABV P-Pep3, and (d) RABV P-Pep4 complexes after MD simulation including polar contacts derived from PyMOL.

Analysis of the RABV P-peptide complexes using the LIGPLOT program indicated the residues involved in the interaction of four designed peptides and RABV P after 100 ns of simulation **(**Supplementary Figure S5**).** LIGPLOT results showed the presence of hydrogen, hydrophobic, and electrostatic bonds in the interaction of Pep2, Pep3, and Pep4 with RABV P, whereas only hydrophobic bindings were indicated in Pep1 and RABV P interaction. Residues in the interaction of RABV P-peptides (Pep1-4) after MD simulation based on LIGPLOT results are listed in Table 5. In comparison between two lists of interface residues in each RABV P-peptide (Pep1-3) complex before and after MD simulations, many common interacting residues could be seen however, there were no observed common interacting residues in the RABV P-Pep4 complex before and after MD simulation. LIGPLOT showed that Pep1 interacts with three key residues from the LC8-binding site of the RABV P including Glu142, Thr146, and Gly150. Glu124, Arg151, and Glu152 from RABV P are also involved in this interaction. Pep2 interacts with five key residues from the LC8-binding site of the RABV P including Glu142, Ser145, Thr146, Thr149 and Gly150. Residues including Gln120, Glu123, Glu124, Arg151, and Glu152 are other five residues from non LC8-binding site involved in this interaction. Pep3 interacts with three key residues from the LC8-binding site including Glu142, Ser145, and Thr146. Residues including Lys116, Gln40, Gln120, Glu124, Pro43, Gly41, and Glu42 from non LC8-binding site, were also participated in this interaction. In RABV P-Pep4 interaction no key residues from LC8-binding site were seen, whereas Asn35, Arg113, Gln36, Glu33, Met108, Glu112, ser110, Arg109, and Gly111 could be seen from non LC8-binding site of RABV P. Interfaces of the RABV P-peptide (Pep1-4) complexes after MD including polar contacts (derived from PyMOL) are presented in Fig. 10a–d.

**Table 5:** Residues in the interaction of RABV P-peptide (Pep1-4) after MD simulation.

	RABV P	Peptide
Residues in the interaction of RABV P-Pep1	Glu124, Glu142, Thr146, Gly150, Arg151, Glu152	Arg60, Asn61, Thr67, His68, Glu69, Thr70
Residues in the interaction of RABV P-Pep2	Gln120, Glu123, Glu124, Glu142, Ser145, Thr146, Thr149, Gly150, Arg151, Glu152	Arg60, Asn61, Phe62, Gly63, Ser64, Tyr65
Residues in the interaction of RABV P-Pep3	Gln40, Gly41, Glu42, Pro43, Lys116, Gln120, Glu124, Glu142, Ser145, Thr146	Arg60, Asn61, Phe62, Gly63, Ser64, Tyr65, His68
Residues in the interaction of RABV P-Pep4	Glu33, Asn35, Gln36, Met108, Arg109, Ser110, Gly111, Glu112, Arg113	Arg60, Asn61, Phe62, Gly63, Ser64, Tyr65, His68, Glu69, Thr70

The underlined amino acids are common amino acids in the RABV P-peptide (Pep1-4) complexes before and after MD simulation.

The average free energy of the RABV P-Pep1, RABV P-Pep2, RABV P-Pep3, and RABV P-Pep4 complexes were −36, −41.02, −27, and −30.36 kcal/mol, respectively. Therefore, The RABV P-Pep2 interaction with the more negative score which owned the highest number of key a.a from RABV P LC8-binding site, exhibited more favorable binding compared to the other three complexes.

## 4 Discussion

The recent attempts at using therapeutic strategies to combat rabies have either not been successful or are still inconclusive [[5], [6], [7], [8], [9], [10], [11], [12], [13], [14], [15], [16],^24^]. The design of structure-based competitive peptides that interfere with virus-host PPIs with significant roles in the viral life cycle, could be a promising therapeutic strategy to combat viral infections [[19], [20], [21]]. The rational knowledge about viral proteins and host partners helps to select the target protein(s) [17]. Among peptides designed based on the function and structure of virus-associated proteins, myrcludex B and T20 could be mentioned [64,65]. Myrcludex B is an N-terminal myristate modification of the first 47 amino acid sequences of the HBV large envelope protein which competes with Hepatitis B virus (HBV) for the sodium-taurocholate co-transporting polypeptide (NTCP; HBV-specific receptor) resulting in HBV-specific receptor blockade and inhibition of viral entry [64]. T20 (enfuvirtide), another peptide drug derived from the C-terminal heptad repeat region of Human Immunodeficiency virus-1 (HIV-1) gp41, is the membrane fusion inhibitor that inhibits HIV entry [65]. In recent decades, a number of RABV-host PPIs have been identified that are required for different steps of the viral life cycle such as viral entry, replication, and spread of virus [23]. Viral-host PPIs, which are involved in viral entry into the host cells, could be the first and the best choice for the design of antiviral peptides since the primary step of the viral life cycle namely viral entry, is inhibited. Indeed, different therapeutic strategies often target viral entry step to limit the infection. (reviewed in Ref. [17]). In the case of RABV, at least four neurospecific receptors have been identified [23], posing challenges for designing antiviral peptides to inhibit viral entry. Therefore, key intraneuronal RABV host PPIs, which are essential for virus replication, budding, and spread, could be alternative targets. To our knowledge, there is no report on the development of inhibitory peptide(s) targeting the key RABV-host PPI(s).

Therefore, we decided to design the inhibitory peptide(s) which could potentially target the RABV P-LC8 interaction, *in silico*. LC8 is an essential factor for RABV transcription and replication. Therefore, this PPI could be a worthy target for the design of a peptide against this virus. For this purpose, molecular docking of RABV P and LC8 followed by MD simulation of the complex was performed to design and select the best inhibitory peptide(s) based on *in silico* analyses. The results showed a stable binding pocket for the MD simulated RABV P-LC8 complex, which fortunately retained most of the key amino acid residues belonging to the binding site of RABV P with human LC8, as also previously introduced [[26], [27], [28], [29],^31^]. A detailed study of the interaction between the LC8 protein and RABV P showed the presence of residues including Arg60, Phe62, Gly63, Ser64, Tyr65, Val66 and Thr67, which belong to the eleven consecutive amino acid containing segment (residues: 60–70) of the LC8 binding region. Four peptides (Pep1, Pep2, Pep3 and Pep4) were designed according to this region and the key amino acids in the interaction between the LC8 protein and RABV P. The 3D structure of these peptides was modeled using the PEP-FOLD3 web server. Designed short peptides were derived from the β-sheet structured hot segment of the binding region of LC8 for its ligands including RABV P. Their interaction as antiviral peptides with RABV P was evaluated using the HADDOCK web server. The stability of these interactions was analyzed using MD simulation. Molecular docking analysis of MD simulated Pep1-4 with RABV P revealed that the RABV P-Pep1 complex had the highest binding affinity and the lowest binding energy. Given that the Pep1 sequence was the complete β-sheet structured hot segment of the binding region of LC8, this achievement seems reasonable. However, after MD simulation of RABV P-peptide (Pep1-4) complexes, Pep2 showed interaction with the higher number of key amino acids from RABV P LC8-binding site compared with Pep1 and RABV P-Pep2 complex indicated the most negative free binding energy score among all. The RMSD results show that RABV P-Pep1 and RABV P-Pep4 complexes fluctuates up to 50 ns and then becomes stable. However, RABV P-Pep2, and RABV P-Pep3 complexes are stable during the simulation. Checking the compression of the complexes using Rg showed that the RABV P-Pep4 complex fluctuated by less than 0.1 nm during the simulation and was stable. The compression of the other peptides was also stable. In order to understand the flexibility in the amino acid region of the RABV P in complex with four peptides, RMSF of RABV P-peptide complexes were calculated. Most of the fluctuations are related to the initial parts of RABV P at the N-terminus (in particular residues 50–70) and at the C-terminus (in particular residues 180–190), which are mostly part of the loop regions. RABV P-Pep1 and P-Pep4 showed higher fluctuations in these two regions than RABV P-Pep2 and RABV P-Pep3. These observations suggest that these two regions of the RABV P-Pep1 and RABV P-Pep4 complexes are less stable and more flexible than other complexes. Also, The SASA analysis results showed that the RABV P-Pep1, RABV P-Pep2, RABV P-Pep3 and RABV P-Pep4 complexes showed similar SASA trends throughout the simulation time with no fluctuation, indicating that there is no change in the exposure to the solvent surface for these complexes. To confirm the simulation results, we ran the simulation for each RABV P-peptide complex with three repetitions. The simulation runs confirmed the reproducibility of the results. The binding free energy indicates the strength of the ligand-receptor interaction. In this study, the binding free energy between RABV-P-peptide complexes was investigated. Analysis of the results showed that the strongest binding with the lowest average free energy was associated with RABV P-Pep2 with an average binding free energy of −41.02. The average free energy of the RABV P-Pep3, and RABV P-Pep4 complexes was more positive which might be due to peptide designing. Since these two peptides were noncontinues sequences of the complete β-sheet structured hot segment of the binding region of LC8, maybe their folding has affected their binding ability to RABV P.

## 5 Conclusion

The results of this study have provided potential candidates for further development of therapeutics against RABV infection. Using *in silico* analysis, we have designed four potential inhibitory peptides to disrupt the RABV P-LC8 complex, with Pep2 showing the highest binding capacity for the RABV phosphoprotein. Pep2 interacts with five key residues from the LC8 binding site of RABV P including Glu 142, Ser 145, Thr 146, Thr 149, and Gly 150. Further *in vitro* and *in vivo* studies will reveal the therapeutic potential of our designed peptides against rabies infection.

## CRediT authorship contribution statement

**Saman Rahmati:** Writing – review & editing, Software, Project administration, Methodology, Investigation, Formal analysis, Data curation. **Fatemeh Zandi:** Writing – original draft, Project administration, Investigation, Funding acquisition, Data curation, Conceptualization. **Khadijeh Ahmadi:** Writing – review & editing. **Ahmad Adeli:** Writing – review & editing. **Niloofar Rastegarpanah:** Software, Formal analysis. **Massoud Amanlou:** Writing – review & editing. **Behrouz Vaziri:** Writing – review & editing, Supervision.

## Ethics approval and consent to participate

Not applicable.

## Data availability statement

The datasets used and/or analyzed during the current study are available from the corresponding author upon reasonable request.

## Funding

This study was supported by grant No. 1356 from 10.13039/501100010679Pasteur Institute of Iran, Iran.

## Declaration of competing interest

The authors declare that they have no known competing financial interests or personal relationships that could have appeared to influence the work reported in this paper.

## List of abbreviations

RABV: Rabies virus
MD: Molecular dynamics
PPIs: Protein-protein interactions
CNS: Central nervous system
HBV: Hepatitis B virus
RMSD: Root mean square deviation
RMSF: Root mean square fluctuation
Rg: Radius of gyration
SASA: Solvent accessible surface area
DSSP: Secondary structure
MM/PBSA: Mechanics Poisson-Boltzmann Surface Area
