# Supplementary material for: Computational structure-based design of antiviral peptides as potential protein–protein interaction inhibitors of rabies virus phosphoprotein and human LC8
Source: Heliyon. 2024 Dec 26;11(1):e41520. doi: 10.1016/j.heliyon.2024.e41520 (PMC11750543; doi:10.1016/j.heliyon.2024.e41520)
Supplement: Multimedia component 1 [file mmc1.docx]

Supplementary Document


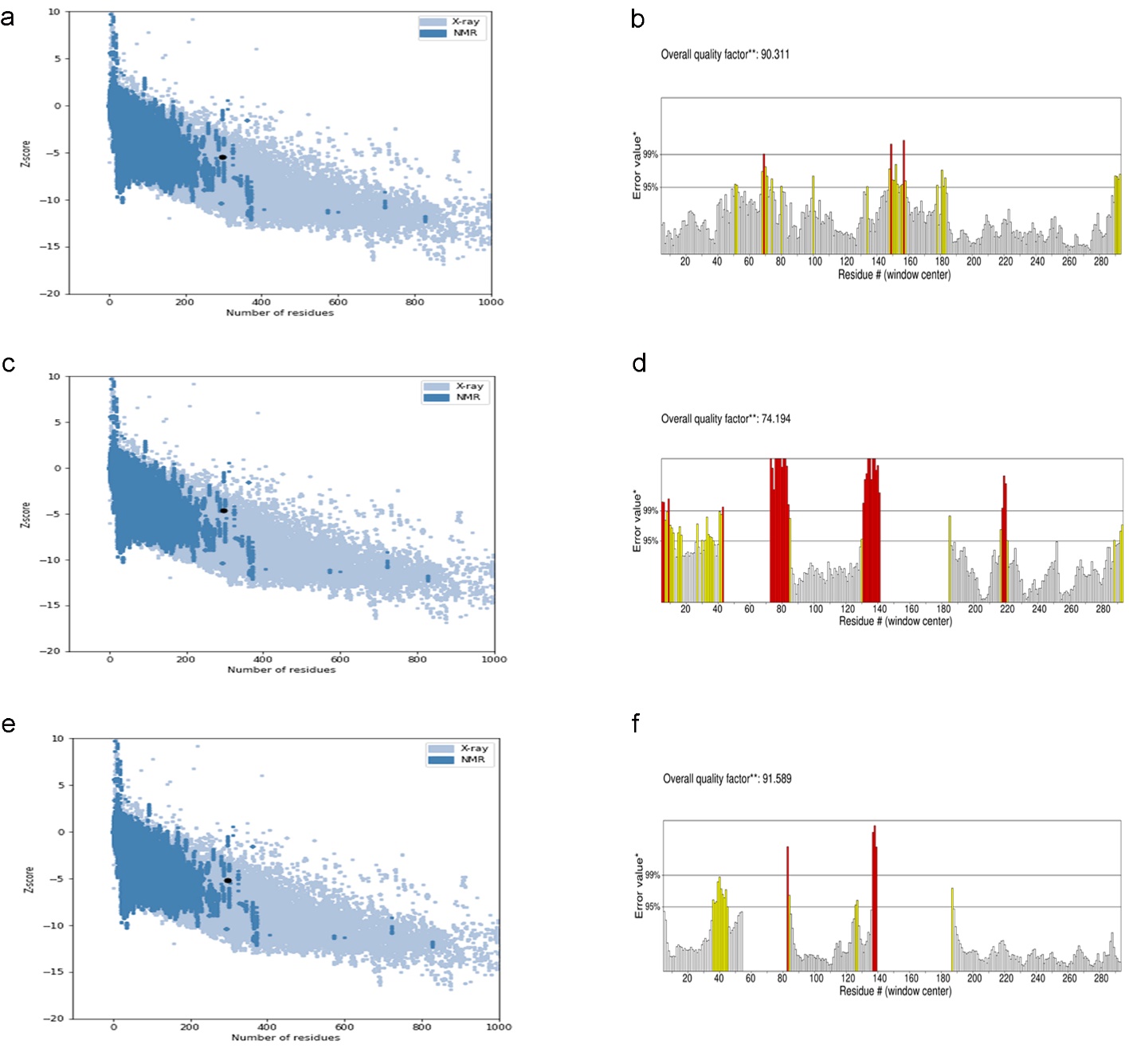


**Supplementary Figure S1.** Structural verification of the generated RABV P model. (a) The ProSA result of the modeled RABV P by I-TASSER compared with the proteins with available crystal/NMR structures. The black dot in the plot shows the Z-score of the modeled RABV P which is located in the range of X-ray and NMR structures. (b) The ERRAT score of the generated model by I-TASSER (c) The ProSA result of the modeled RABV P by MODELLER compared with the proteins with available crystal/NMR structures. The black dot in the plot shows the Z-score of the modeled RABV P which is located in the range of X-ray and NMR structures. (d) The ERRAT score of the generated model by MODELLER. (e) The ProSA result of the modeled RABV P by AlphaFold2 compared with the proteins with available crystal/NMR structures. The black dot in the plot shows the Z-score of the modeled RABV P which is located in the range of X-ray and NMR structures. (f) The ERRAT score of the generated model by AlphaFold2.


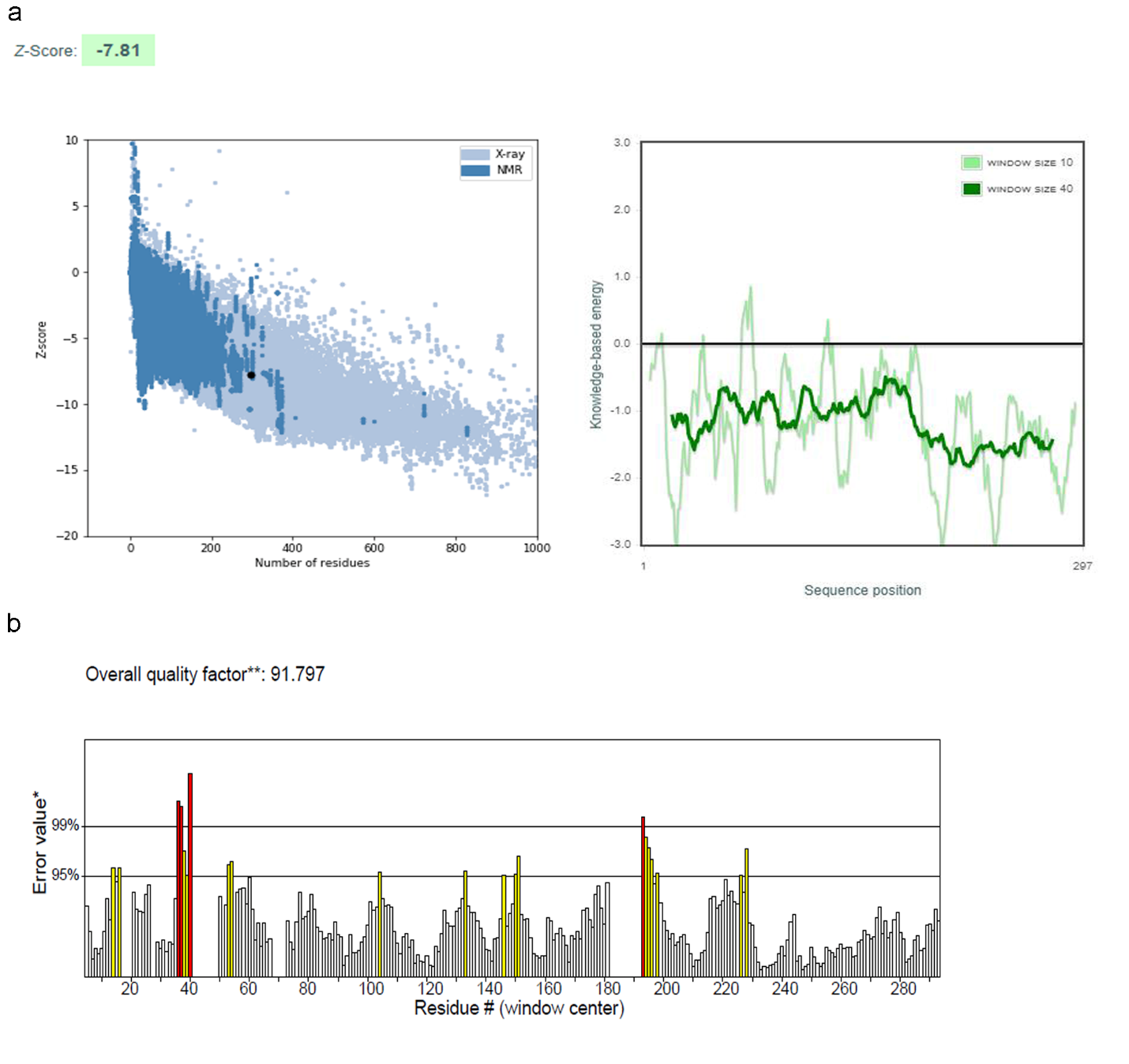
**Supplementary Figure S2.** Validation of the MD simulated RABV P. (a) The ProSA result of post MD RABV P compared with the proteins with available crystal/NMR structures. The black dot in the plot shows the Z-score of the modeled RABV P which is located in the range of X-ray and NMR structures (left figure). The negative energy plot indicates no problematic amino acid residue in the model (right figure). (b) The ERRAT score of the post MD modeled RABV P indicates the acceptable quality of the structure.


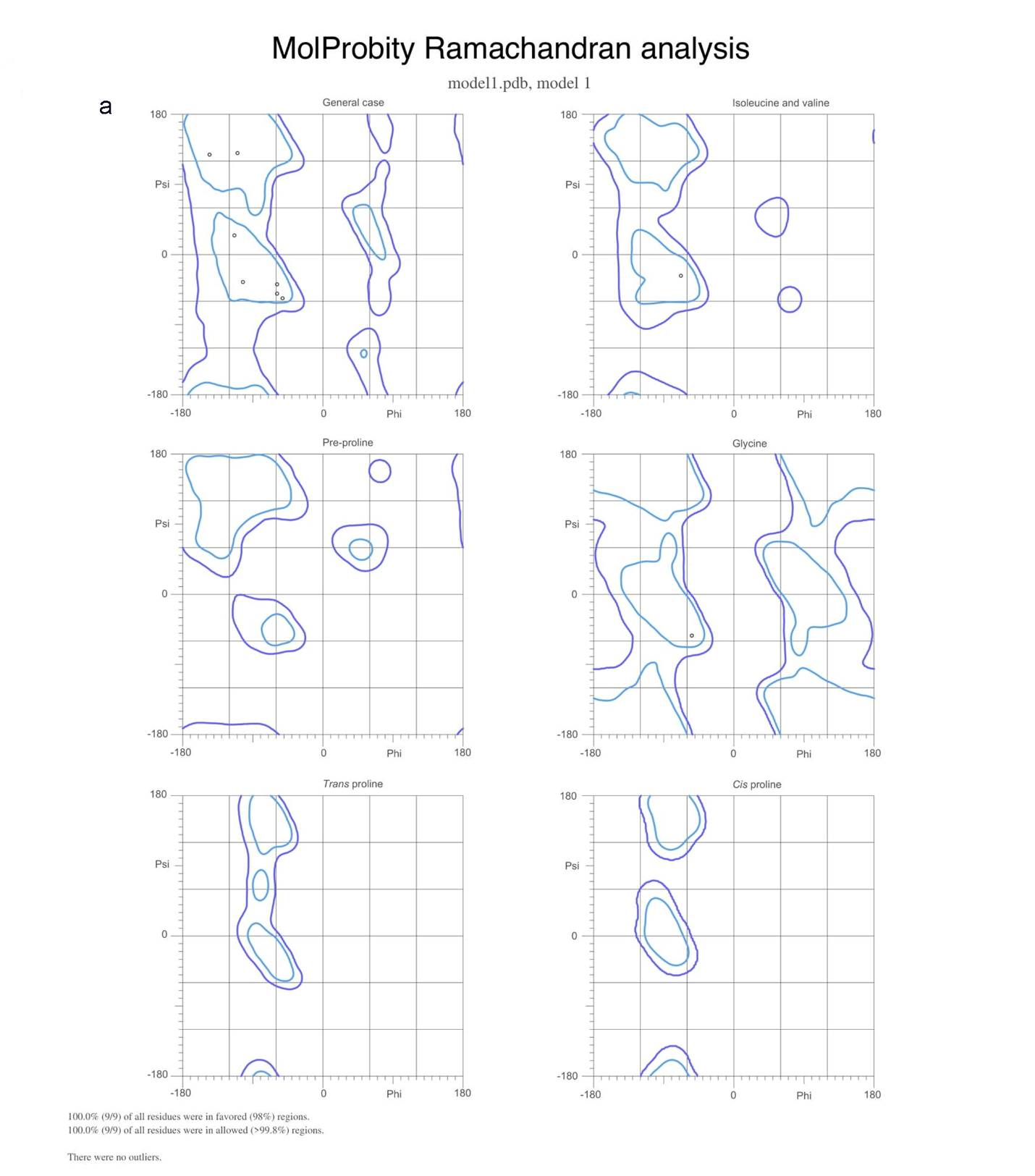


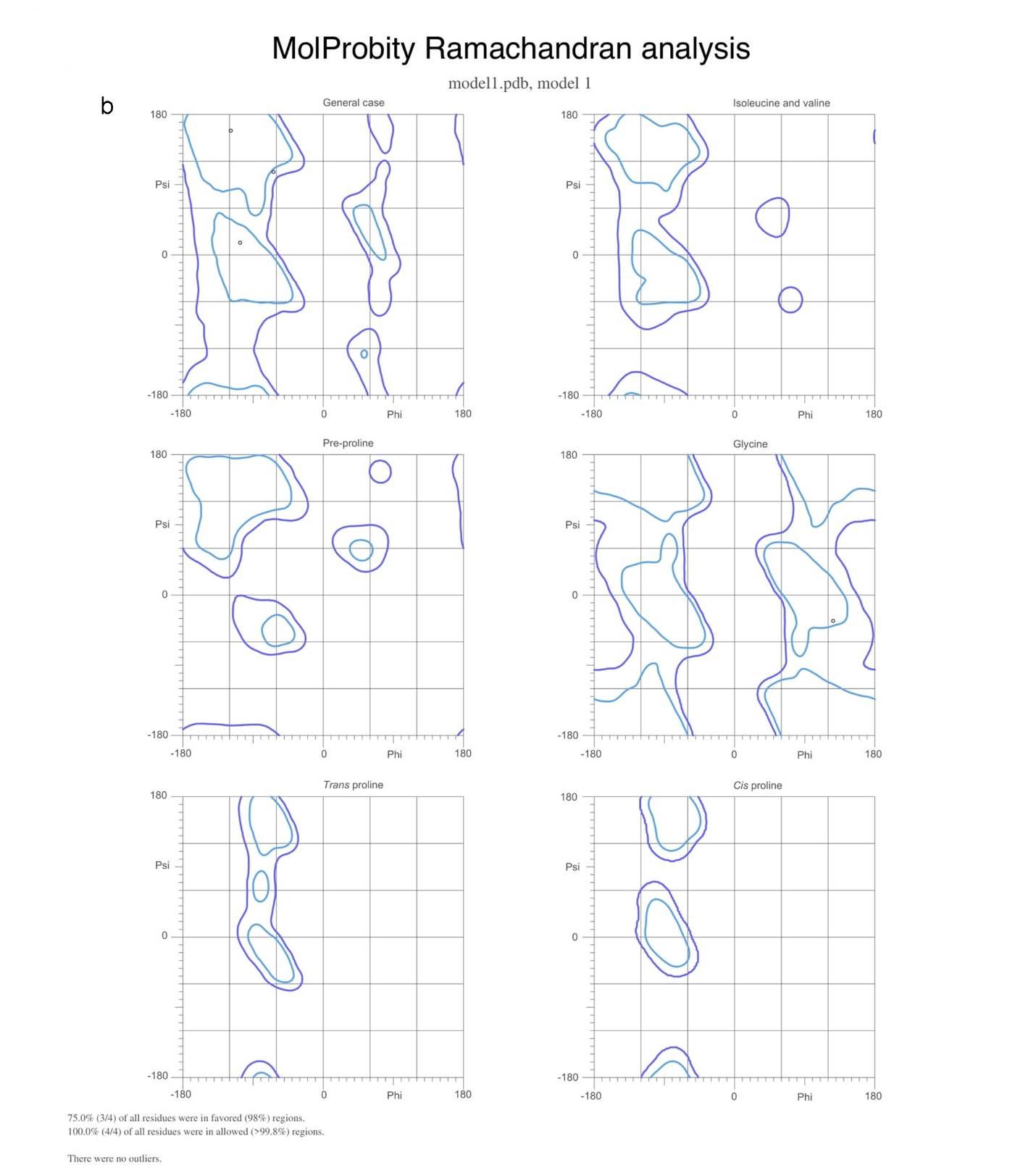


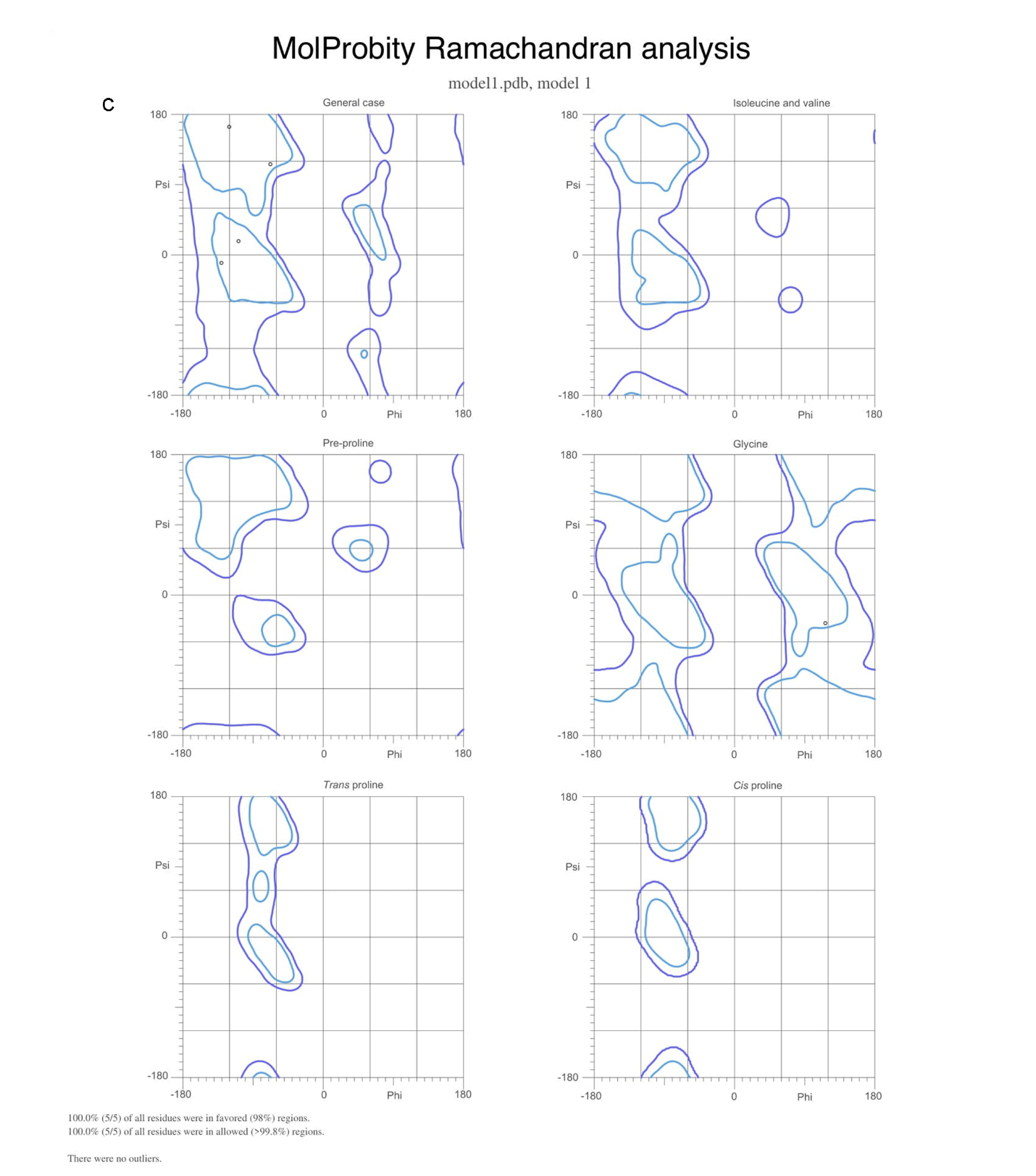


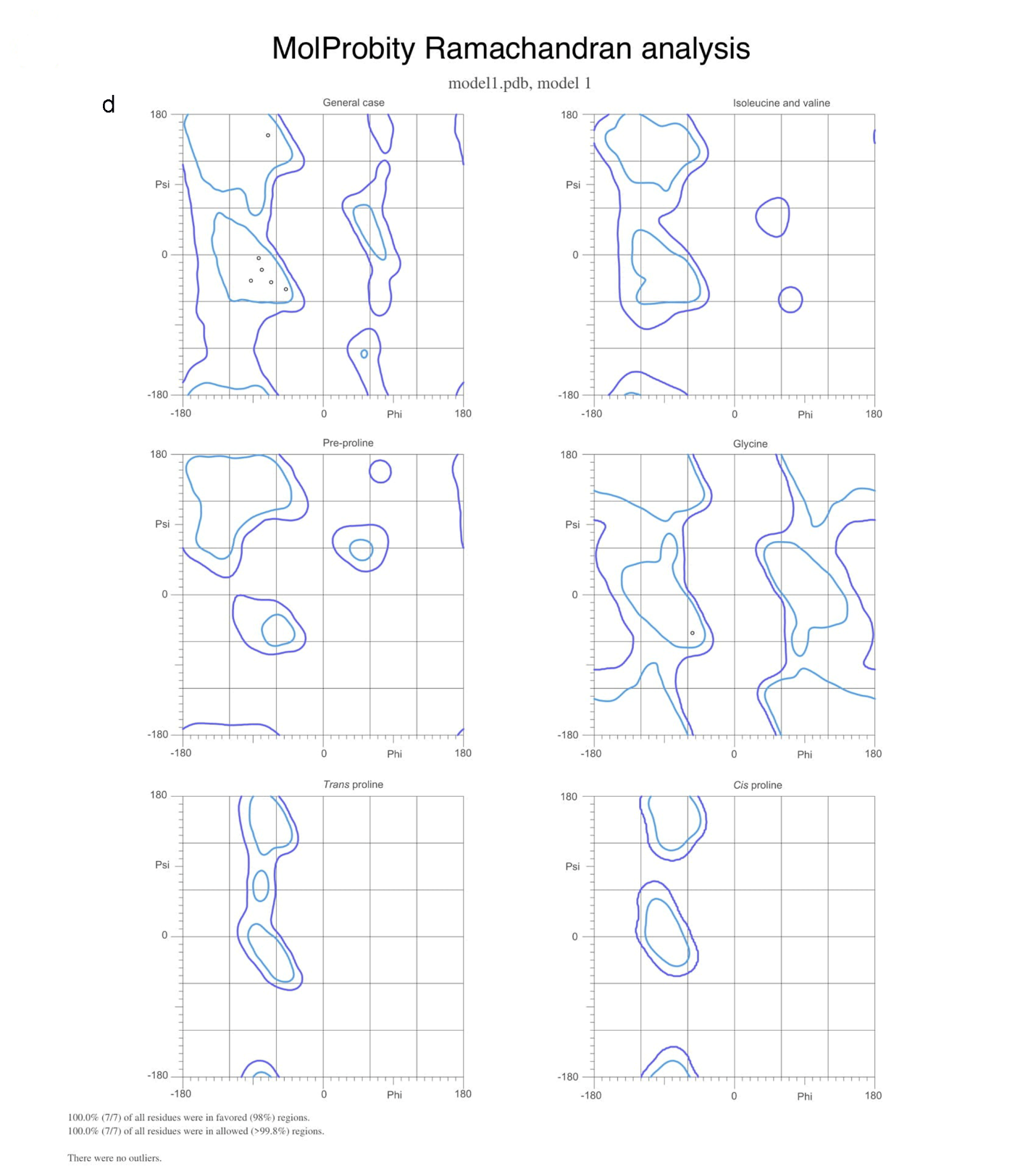


**Supplementary Figure S3.** Evaluation of the modelled peptides using the Ramachandran plot. (a) There was no residue of Pep1 in the outlier region. (b) There was no residue of Pep2 in the outlier region. (c) There was no residue of Pep3 in the outlier region. (d) There was no residue of Pep4 in the outlier region.

**Supplementary Table S1.** Characterization of the proinflammatory antigenicity and toxicity of the designed peptides using the Proinflam and the ToxinPred web servers, respectively. (a) The proinflammatory antigenicity situation of Pep1 was negative and it was characterized as non-toxic. (b) The proinflammatory antigenicity situation of Pep2 was negative and it was characterized as non-toxic. (c) The proinflammatory antigenicity situation of Pep3 was negative and it was characterized as non-toxic. (d) ) The proinflammatory antigenicity situation of Pep4 was negative and it was characterized as non-toxic.


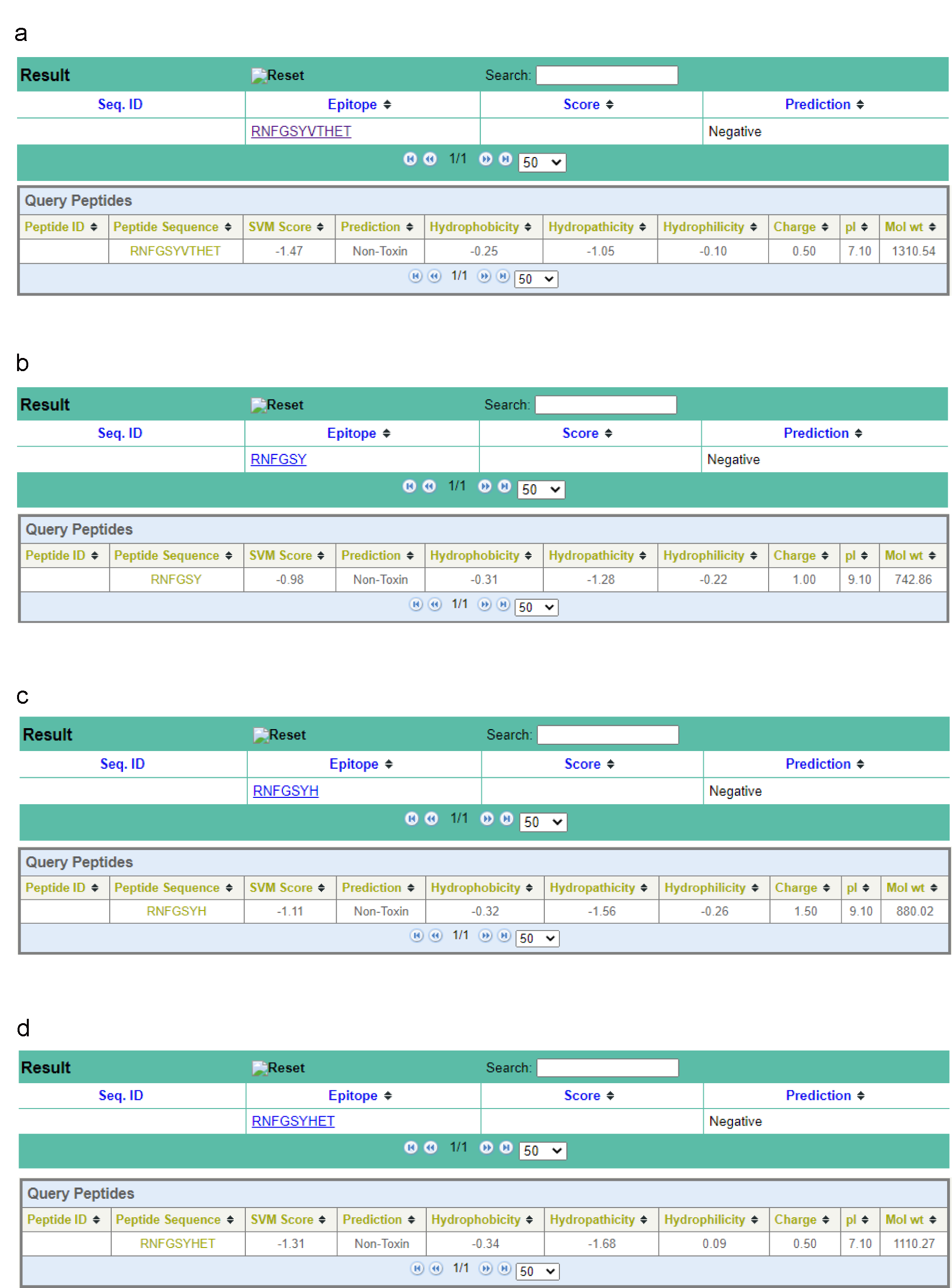


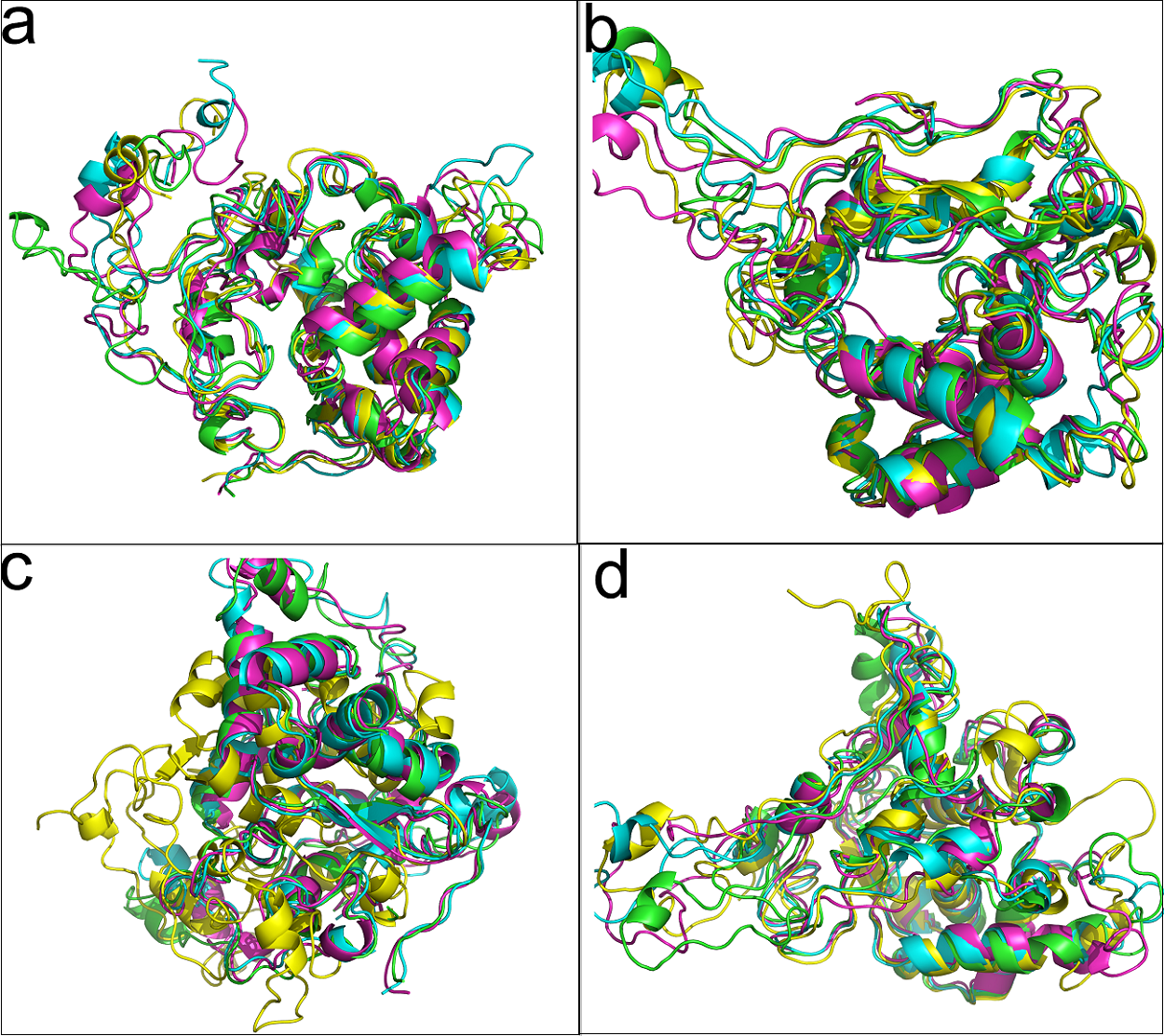


**Supplementary Figure S4.** Snapshots of 25, 50, 75 and 100 ns of MD simulation of the RABV P-peptide complexes. Yellow: 25 ns, blue: 50 ns, purple: 75 ns, light green: 100 ns. (a) RABV P-Pep1 complex, (b) RABV P-Pep2 complex, (c) RABV P-Pep3 complex, (d) RABV P-Pep4 complex.


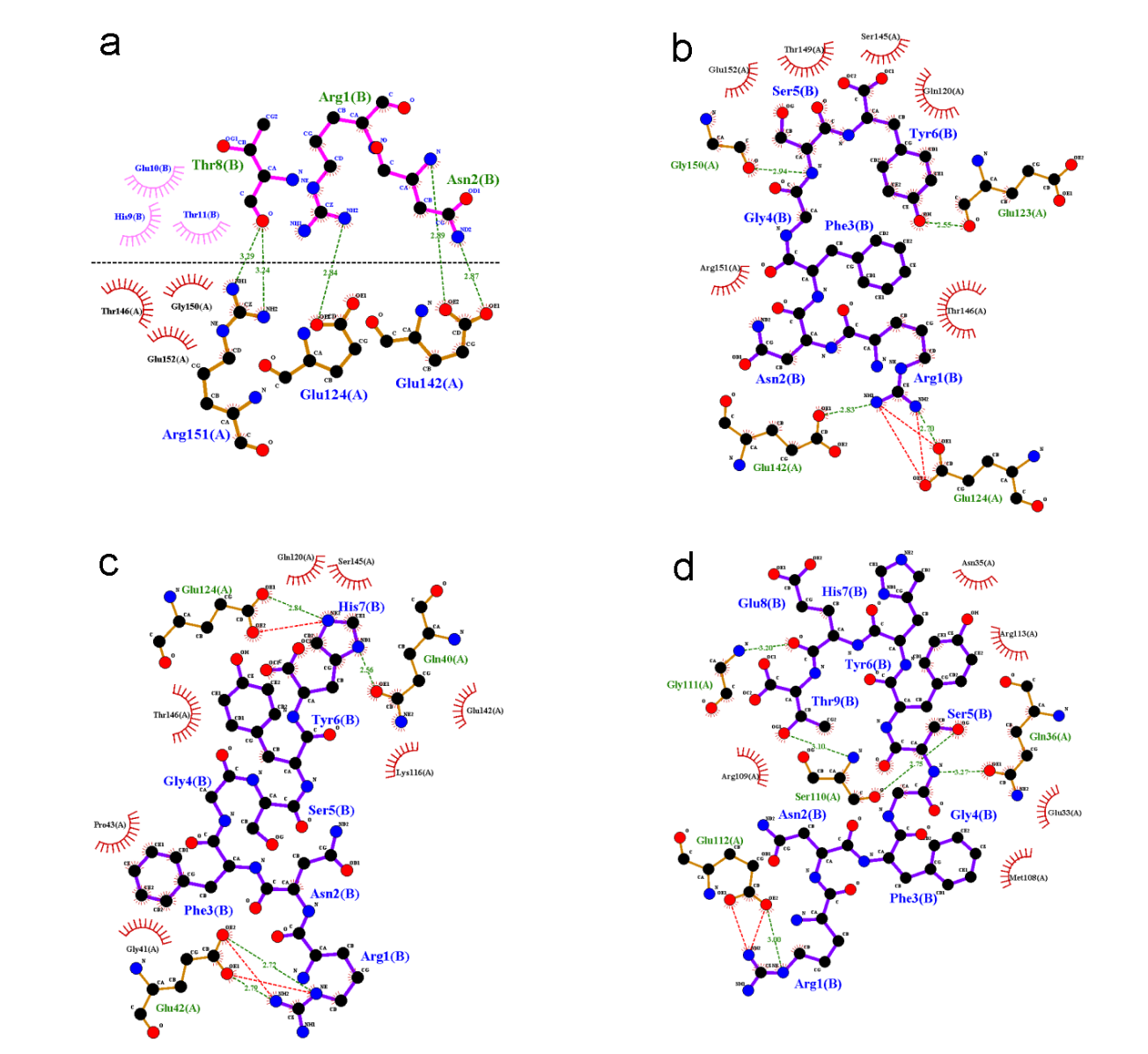


**Supplementary Figure S5.** 2D-LIGPLOT diagrams indicating interacting amino acid residues in (a) RABV P-Pep1, (b) RABV P-Pep2, (c) RABV P-Pep3, (d) RABV P-Pep4 post MD simulated complexes. The amino acid residues at RABV P interface (chain A) and peptides (Pep1-4) interfaces (chain B) involved in hydrophobic interactions are shown as brown and pink spoked arcs, respectively. Side chains of amino acid residues involved in hydrogen bond interactions are shown as ball-and-stick models. Hydrogen and Electrostatic bonds are shown by green and red dotted lines, respectively.
